# Variation in Prices Charged to Patients for Specialty Intraocular Lenses Inserted during Universally Covered Cataract Surgery

**DOI:** 10.1371/journal.pone.0035179

**Published:** 2012-04-24

**Authors:** Joshua M. Robert, Robert J. Campbell, Chaim M. Bell

**Affiliations:** 1 Faculty of Medicine, University of Toronto, Toronto, Ontario, Canada; 2 Department of Ophthalmology, Hotel Dieu Hospital and Queen's University, Kingston, Ontario, Canada; 3 Institute for Clinical Evaluative Sciences, Toronto, Ontario, Canada; 4 Departments of Medicine and Health Policy Management and Evaluation, University of Toronto, Toronto, Ontario, Canada; 5 Department of Medicine, Keenan Research Centre, The Li Ka Shing Knowledge Institute of St. Michael's Hospital, Toronto, Ontario, Canada; Medical University Graz, Austria

## Abstract

**Background:**

Patients often pay for specialty intraocular lenses (IOLs) for cataract surgery covered by universal insurance. This practice creates the potential for inequitable pricing where the medical service provider is also the retailer. We measured the variation in prices between cataract surgeons for the same IOL and associated testing.

**Methods:**

We telephoned every cataract surgeon in Ontario, Canada, and asked their price for the most common type of specialty IOL as a prospective patient. We measured the total prices quoted and variation between providers.

**Results:**

We contacted 404 ophthalmologists. There were 256 that performed cataract surgery but 127 offered the most commonly employed specialty IOL and would provide a price to patients over the telephone. We obtained prices from all 127 ophthalmologists. Prices for the same lens and associated testing varied substantially between ophthalmologists from $358 to $2790 (median $615, interquartile range $528–$915). There was variation in all components of the total out-of-pocket price, including the price for the IOL itself, charges for uninsured eye measurements, and non-specific supplemental fees.

**Conclusion:**

Although cataract surgery is covered by public health insurance, some ophthalmologists charge much more than others for the same specialty IOL and associated testing. Greater access to price information and better regulatory control could help ensure patients receive fair value for out-of-pocket health expenses.

## Introduction

Cataract surgery is a common procedure that affords patients improvements in both visual acuity and quality of life [1]. During the surgery, the opacified natural lens is removed and an artificial intraocular lens is inserted. The procedure is fully insured by universal coverage throughout Canada. The timely provision of cataract surgery across Canada has been a focus of politicians and physicians as a priority service of the Wait Time Alliance [2].

Advances in intraocular lens (IOL) technology have brought new options to patients undergoing cataract surgery. Specialty IOLs—comprising accommodating, multifocal, and toric types—offer patients the possibility of refractive correction at the time of cataract surgery [3]. Although these IOLs may offer advantages and convenience to specific sub-groups of patients, specialty IOLs have been deemed not “medically necessary” by most payors. Hence, the additional costs of these lenses are not covered by most provincial insurance plans and must be paid for by patients [4].

This situation—where patients pay out-of-pocket for “specialty options” within an insured service—creates issues which are germane to patients and policymakers. In the absence of regulation, the prices patients are charged may be inequitable because individual physicians are free to set their own fees for uninsured services. While guidelines on pricing exist, these remain voluntary and the degree of uptake is unknown. Substantial price variation between surgeons for equivalent specialty lenses would represent a market failure which might warrant regulatory intervention.

We conducted a study of all cataract surgeons in Ontario, Canada to measure the variation in the total out-of-pocket price for a specialty IOL from the patient perspective.

## Methods

### Identifying Cataract Surgeons

We first searched the online public register of the College of Physicians and Surgeons of Ontario (CPSO) for all registered ophthalmologists as of June 2010 [5]. We supplemented our CPSO-generated list with a search of the online Canadian Ophthalmological Society (COS) directory for all Ontario members to ensure completeness. We excluded resident trainees, clinical fellows, and ophthalmologists with no practice address in Ontario, as well as those whose sole practice location was at a pediatric facility.

### Choice of Specialty Lens Type

We selected one type of specialty lens for consistency of comparisons. We chose the toric type of specialty IOL because it is one of the most commonly used specialty lenses in Canada [6], and one manufacturer (Alcon AcrySof Toric model by Alcon Laboratories, Inc., Mississauga, Ontario, Canada.) dominates the market in Canada [3].

### Eliciting Specialty Lens Prices

Our telephone survey was designed to obtain the price information that a potential patient would receive. We collected data by telephoning all ophthalmologists' primary practice locations identified by our search. We conducted brief, structured telephone interviews with clinic staff, between June and October, 2010 (Telephone Interview Script S1). We did not disclose our research intent, since this may have reduced our response rate. When staff members at an ophthalmologist's primary practice were unavailable or unable to answer our questions, we followed up with secondary practice locations. We asked about all associated charges, such as uninsured eye measurements and extra office fees. For example, the eye measurements employed by surgeons for pre-operative diagnostic imaging must often be paid for out-of-pocket.

We repeated phone interviews with fifteen (12%) randomly chosen surgeons' offices to assess the reproducibility of our results at least six weeks after the original interview. We did not make any reference to our original conversation when calling for a repeat quote.

### Data Analysis and Statistics

Data were tabulated and standardized for comparison by calculating a total out-of-pocket price. All prices were determined per eye, rounded to the nearest dollar before tax for the entire package of specialty lens and associated eye measurements. Data were analyzed using descriptive statistics. To quantify the variation in prices, we calculated the coefficient of variation (CV), which is defined as the standard deviation divided by the mean.

### Ethics Approval

The interviewer did not disclose research intent for the study. They did not ask for individual informed consent from the respondents because we felt that this might bias or alter the responses. Our study protocol was approved by the St. Michael's Hospital Research Ethics Board, Toronto, Canada.

## Results

We identified 517 ophthalmologists registered with the CPSO as of June 16, 2010. Of these, 404 ophthalmologists met our inclusion criteria and were contacted. There were 356 ophthalmologist offices willing to provide a response. Of these, 256 performed cataract surgery and 127 offered the type of IOL we were studying. We obtained a price quote from all 127 ophthalmologists (Table 1). This corresponded to an 88% (356/404) response rate for eligible ophthalmologists and a 100% (127/127) response rate for offices who would offer a price over the phone for the lens we sought.

**Table 1:** Numbers of ophthalmologists identified, phoned, and interviewed.

	Number	Group or Sub-Group
	517	Ophthalmologists registered with CPSO
-	113	Ophthalmologists excluded: no practice address in Ontario, practice exclusively at a pediatric facility, clinical fellows, resident trainees.
=	404	Ophthalmologists' offices phoned
-	148	Ophthalmologists who do Not perform Cataract Surgery (NCS)
=	256	Cataract surgeons
-	81	Cataract surgeons who do Not offer a Toric Lens (NTL)
-	32	Office staff did not know prices and were unwilling to follow-up
-	16	Cataract surgeons who will only quote prices during an office consultation
=	127	Price Quotes Obtained (PQO), 108 itemized price quotes & 19 lump-sum quotes.
		Total ophthalmologists' offices interviewed = (NCS+NTL+PQO) = 356

**Figure 1:**
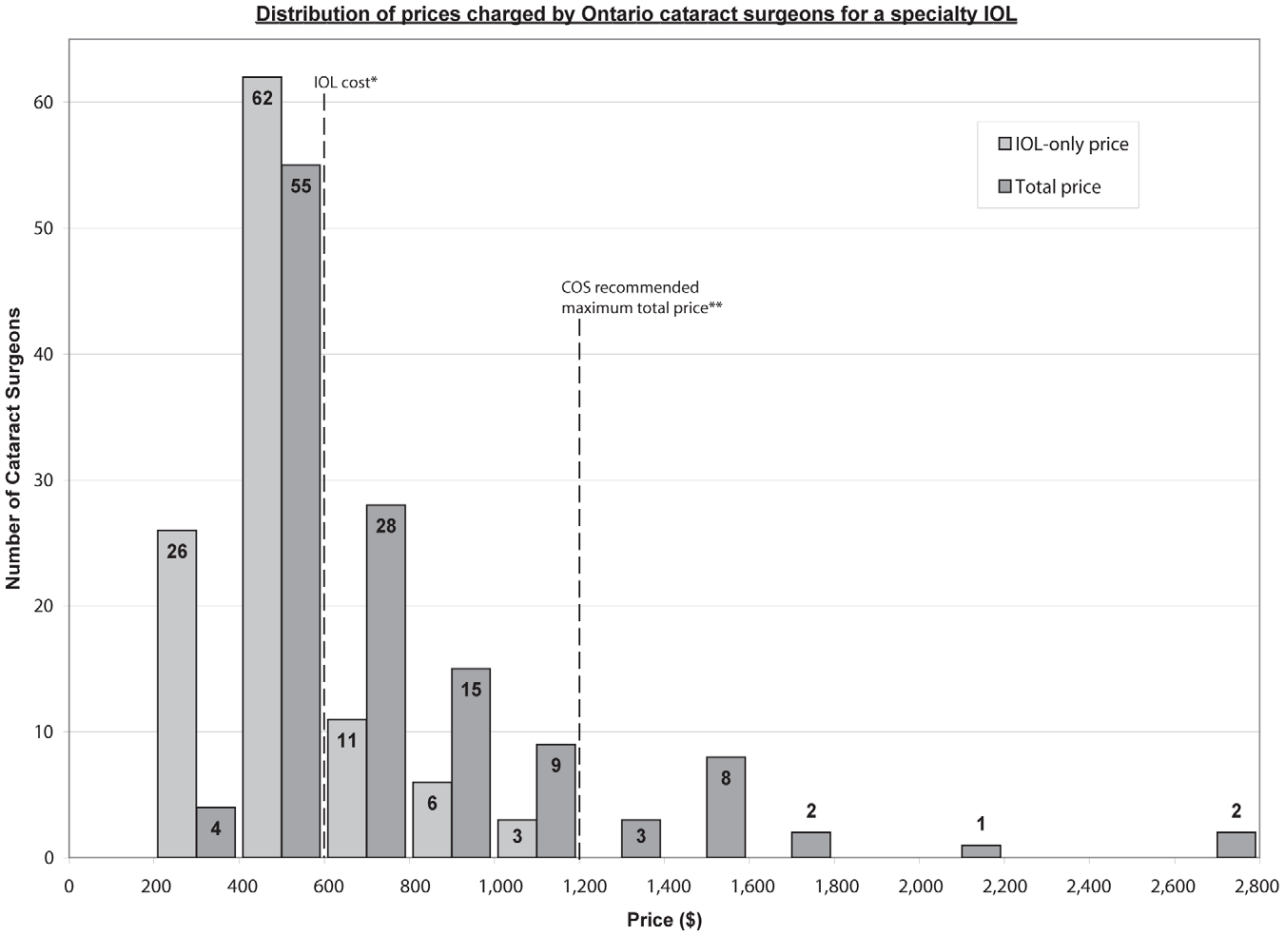
Distribution of prices charged by Ontario cataract surgeons for a specialty IOL. IOL-only prices represent the price quoted for a single IOL, before any additional fees. IOL-only prices are presented for surgeons who provided itemized price quotes (n = 108). Total prices are calculated as total per eye for IOL plus all associated eye measurements and extra fees. Total prices are presented for all surgeons who provided itemized or packaged price quotes (n = 127). *IOL cost: $550. **COS recommended maximum total price: $1,144. IOL: Intraocular Lens. COS: Canadian Ophthalmological Society.

The median price for a specialty IOL, including all associated fees, was $615 (interquartile range $528 to $915). Prices ranged from $358 to $2790, with a CV of 0.53. The distribution of prices is presented in Figure 1. For the 108 ophthalmologists who provided a specific price for the lens, this value ranged from $270 to $1200, with a median of $450. Our structured interview accounted for the possibility that different cataract surgeons may offer equivalent, but not identical IOLs (i.e. toric lenses from competing manufacturers). However, we found that every price quote we obtained was for the exact same IOL, the Alcon Toric model.

Charges for the non-lens component of the total fee varied considerably between surgeons. These extra charges could only be analyzed for the 108 ophthalmologists who provided itemized pricing. Extra charges fell into one of two categories: charges for specific uninsured diagnostic imaging tests, and non-specific supplemental office/administrative fees. The median charge for eye measurements was $100 (range $0–$500, N = 108). For most surgeons, this fee was for IOLMaster laser biometry (Carl Zeiss Canada Ltd., Toronto, On.). Some surgeons also required and charged for other measurements (e.g. corneal topography, optical coherence tomography). Higher charges did not always apply to more extensive testing. Nine surgeons charged a non-specific supplemental fee, which ranged from $50 to $600 (median $230), in addition to a measurement fee. Of the seven surgeons who charged only for the IOL, two utilized the OHIP-insured A-scan ultrasound measurement, and five offered the IOLMaster free-of-charge.

For the fifteen repeat interviews, eleven of the repeat quotes were identical to the original. For the remaining four, the mean difference between original and repeat price quote was $49 (range $20 to $100).

## Discussion

We contacted every cataract surgeon in Ontario and asked their price for the most common type of specialty lens implanted during cataract surgery. We found that out-of-pocket prices for specialty lenses varied almost eight-fold between ophthalmologists for the same product. As well, there was substantial variation in the non-lens component of the fees charged. We also discovered that some cataract surgeons do not discuss prices over the phone.

Our study is unique because we have compared the specialty IOL prices that a prospective patient would be quoted. Our telephone interviews were simple, and allowed us to obtain an 88% response rate for eligible ophthalmologists and a 100% response rate for offices who discuss prices over the phone. We reduced potential bias by not disclosing our research intent, since this may have reduced our response rate.

We found no similar peer-reviewed data on this topic. A previous non-peer reviewed survey of 54 ophthalmologists from across Canada reported a mean toric IOL price of $657 [4]. No calculations on outliers or measures of variation were included. As well, we are aware of three websites which facilitate price comparison shopping for medical services in the United States: pricedoc.com, newchoicehealth.com, and outofpocket.com. However, data from these sites cannot be compared to our findings because their prices include surgery and facility fees in addition to lens and eye measurement fees.

How does the degree of variation in prices for specialty lenses compare to that of other medical products and services? Previous work using a similar methodology measured variation in charges for elective hospital procedures and generic prescription drugs. An overall coefficient of variation of 0.53 was reported across hospitals in the United States and Canada for prices of selected therapeutic services and a coefficient of variation of 0.16 was reported across pharmacies in Canada for prices of selected generic medications [7], [8]. Our calculated coefficient of variation for Ontario cataract surgeons suggests that the variability in price for a specialty lens is similar to other therapeutic services, but much greater than the variability in generic drug prices.

The COS has established guidelines for charges for uninsured services including specialty lens implants and associated testing. Specifically, the COS recommended maximum total charge for a toric IOL with IOLMaster biometry is $1144, based on a published IOL cost of $550 (the actual cost of the IOL may be less, depending on confidential and individually negotiated contracts between surgeons/hospitals and the manufacturer) [9]. We found that the majority of cataract surgeons charge less than the recommended maximum total price, but some surgeons charge up to thousands of dollars more. As well, as evident in Figure 1, many surgeons' total prices are less than the higher charging surgeons' IOL-only prices. The implications of this finding are pertinent to any patient considering a specialty lens: comparison shopping may offer substantial savings. But comparison shopping for a specialty lens is not a simple task. To collect data, we had to make repeated calls to surgeons' offices and we had to be insistent when our request for a price quote was met with the response “you'll have to make an appointment to see the doctor.” Although some surgeons may assert that they can only answer price questions after they have assessed the patient and determined the procedure to be undertaken, having a strict policy of only discussing their prices with patients in-person is a barrier to the provision of open and fair price comparison information.

One economic explanation for the considerable variation in prices is that an informational asymmetry is creating a market failure. For most products, consumer price searching acts as an important force which limits price dispersion [10]. However, it has long been recognized that individuals do not shop for healthcare in that same rational manner that they would for other consumer goods. For example, patients have been found to prefer local over regional surgical centres despite a lower operative mortality risk conferred by regional centres [11]. As well, patients have been found to forego comparing hospitals for major surgery even though the same patients considered surgeon and hospital performance data to be meaningful and relevant [12]. In the prescription drug market, pricing can vary between neighborhoods [13] and reliable price comparisons are difficult to find [14]. Given the large price variation we observed, it would seem the market for specialty lenses is a striking example of the difference between classical and medical economics.

Our study carries special relevance for policymakers. We have identified highly variable pricing for similar and often identical products and services. While most surgeons appear to charge reasonable prices, some have established prices much higher than can be considered reasonable or fair. Since the facility fees and overhead costs of cataract surgery are universally covered by a government payor, those surgeons who set prices at the high end of the range may earn more from the added charges associated with specialty lenses than from performing the operation itself [15]. Potential solutions to protect the patient as consumer in this context would involve making the prices publicly available or regulating prices either through a third party (e.g. the publicly-funded hospital) or through legislation.

Several limitations merit mention. First, although we contacted all cataract surgeons in Ontario, not all responded with their prices. Indeed, the range and variation observed may have been even greater had we been able to obtain information from non-responders. Second, we only asked for the price of one type of specialty lens, but it is likely that the factors determining specialty lens prices do not differ widely between various types of lenses. Third, though we were diligent to ask staff at each office for the total price which patients must pay, it is possible that some fees were not disclosed. Finally, though we surveyed Ontario ophthalmologists, our study is likely generalizable to all jurisdictions where out-of-pocket payment for medical care is permitted.

In many countries it is the exception that physicians act as both health care providers and medical service retailers. The case of cataract surgery with specialty intraocular lenses is a notable example. Our findings suggest that some ophthalmologists charge much more than others for the same service. Greater access to price information and better regulatory control could help ensure patients receive the right care at a fair price.

## Supporting Information

Telephone Interview Script S1 (DOC)

## References

[1] Asbell PA, Dualan I, Mindel J, Brocks D, Ahmad M (2005). Age-related cataract.. The Lancet.

[2] Wait Time Alliance (2010). Still waiting.. http://www.waittimealliance.ca/index.htm.

[3] Slade SG, Mertens EL (2010). The state of the premium IOL market.. Cataract and Refractive Surgery Today Europe January.

[4] Peachey D, Hicks V (2010). Valuation of Uninsured Ophthalmological Services: Report to the Canadian Ophthalmological Society.. http://www.eyesite.ca/english/program-and-services/policy-statements-guidelines/Uninsured-services_COSreport_Apr2010.pdf.

[5] College of Physicians and Surgeons of Ontario (2010). The Public Register.. http://cpso.on.ca/docsearch/default.aspx?id=2048.

[6] Ong-Tone L, Bell A (2010). Practice patterns of Canadian Ophthalmological Society members in cataract surgery—2009 survey.. Canadian Journal of Ophthalmology.

[7] Redelmeier DA, Bell CM, Detsky AS, Pansegrau GK (2000). Charges for Medical Care at Different Hospitals.. Archives of Internal Medicine.

[8] Gooi M, Bell CM (2008). Differences in Generic Drug Prices between the US and Canada.. Applied Health Economics and Health Policy.

[9] Canadian Ophthalmological Society (2010). COS Statement on Values for Uninsured Services in Canada.. http://www.eyesite.ca/english/program-and-services/policy-statements-guidelines/Uninsured-services_COSstatement_Apr2010.pdf.

[10] Telser LG (1973). Searching for the Lowest Price.. The American Economic Review.

[11] Finlayson SRG, Birkmeyer JD, Tosteson AN, Nease RF (1999). Patient preferences for location of care: implications for regionalization.. Med Care.

[12] Schwartz LM, Woloshin S, Birkmeyer JD (2005). How do elderly patients decide where to go for major surgery?. BMJ.

[13] Gellad WF, Choudhry NK, Friedberg MW, Brookhart MA, Haas JS (2009). Variation in drug prices at pharmacies: are prices higher in poorer areas?. Health Serv Res.

[14] Vogel L (2011). Comparison shopping for drugs.. CMAJ.

[15] Ministry of Health and Long-Term Care (2010). Schedule of Benefits for Physician Services under the Health Insurance Act. Queen's Printer for Ontario.. http://www.health.gov.on.ca/english/providers/program/ohip/sob/physserv/physserv_mn.html.

