# Supplementary material for: Variation in Prices Charged to Patients for Specialty Intraocular Lenses Inserted during Universally Covered Cataract Surgery
Source: PLoS One. 2012 Apr 24;7(4):e35179. doi: 10.1371/journal.pone.0035179 (PMC3335842; doi:10.1371/journal.pone.0035179)
Supplement: Telephone Interview Script S1 — (DOC) [file pone.0035179.s001.doc]

**S1.** Telephone Interview Script.

*Hello, I am calling to ask some questions about cataract surgery. Is someone available to answer my questions?*

1. *Does Dr.* *(ophthalmologist’s name) offer the Alcon Toric type of* *lens for cataract surgery*?
   1. If YES: *How much does it cost for that type of lens?* (Continue to 3)
   2. If NO: *Does the doctor offer an equivalent type of toric lens?* (If YES, continue to 2bi; If NO, continue to 2c)
      1. *How much does it cost?* (Continue to 3)
   3. If no toric lenses offered: *Does the doctor do cataract surgery, just not with toric lenses; or does he/she not do cataract surgery at all?* (Continue to 6)
2. *I understand that some eye-measurements are needed to use that lens, how much do the measurements cost*?
3. *Are those prices for one eye, or both*?
4. *Are there any other costs, like an office fee or calculation fee*?
5. *I really appreciate your time, thank you*.
